# Alternative Diagnostic Strategy for the Assessment and Treatment of Pulmonary Embolus: A Case Series

**DOI:** 10.5811/cpcem.2020.5.46517

**Published:** 2020-06-22

**Authors:** Ayaz Aghayev, Aliza A. Memon, Paul Gregg Greenough, Lakshmi Nayak, Sijie Zheng, Andrew M. Siedlecki

**Affiliations:** * Brigham and Women’s Hospital, Department of Radiology, Boston, Massachusetts; † Brigham and Women’s Hospital, Department of Internal Medicine, Boston, Massachusetts; ‡ Brigham and Women’s Hospital, Department of Emergency Medicine, Boston, Massachusetts; § Dana Farber Cancer Institute, Boston, Massachusetts; ¶ Kaiser Permanente, Oakland, California

**Keywords:** FE-MRA, pulmonary embolus, renal transplant, lymphoproliferative

## Abstract

**Introduction:**

Ferumoxytol-enhanced magnetic resonance angiography (FeMRA) can be used as an alternate and safe method to diagnose patients with compromised renal function who present with acute pulmonary embolus in the emergency department (ED) setting.

**Case Report:**

A 62-year old man with a history of renal transplant and lymphoproliferative disease described new onset of breathlessness. His clinical symptoms were suggestive of pulmonary embolus. He underwent FeMRA in the ED to avoid exposure to intravenous iodinated contrast. FeMRA demonstrated a left main pulmonary artery embolus, which extended to the left interlobar pulmonary artery. Afterward, the patient initiated anticoagulation therapy. With preserved renal function he was able to continue his outpatient chemotherapy regimen.

**Conclusion:**

This case highlights a safe imaging technique for emergency physicians to diagnose pulmonary embolus and subsequently guide anticoagulation therapy for patients in whom use of conventional contrast is contraindicated.

## INTRODUCTION

Kidney transplant recipients are frequently evaluated in the emergency department (ED) setting with a visit rate of 1.4 per patient-year.^1^ The majority of these encounters are in the first two years after transplant, which vary based on the center as well as patient characteristics.^2^ Most often, an infectious etiology is identified in the first year post-transplant whereas cardiopulmonary disease and malignancy are detected after the first year of engraftment.^3^,^4^ Patients receiving a solid organ transplant have increased rates of post-transplant lymphoproliferative disease due to chronic immunosuppressive therapy and a dysfunctional immune system.^5^ Renal transplant patients have an eight-fold increased risk of thromboembolism compared to the general population.^6^

These characteristics present a diagnostic challenge for the emergency physician when assessing for pulmonary embolus in kidney transplant recipients. As a result this population is at risk for iodine contrast-induced nephropathy at higher rates than patients with bilateral native kidney function with similar estimated glomerular filtration rate (eGFR)^7^,^8^; however, it is unclear whether this broadly applies to patients with chronic kidney disease (CKD) and a decreased renal reserve in the setting of a solitary kidney transplant since this group is either excluded or under-represented in multivariate analyses.^9^ We report the use of ferumoxytol-enhanced magnetic resonance angiography (FeMRA) as an alternative diagnostic tool to assess for pulmonary embolus in patients at risk of iodine contrast-induced nephropathy. Awareness of alternative imaging techniques in the ED setting for patients with severe CKD or kidney transplant recipients with CKD may offer expedited diagnosis and treatment.

## CASE SERIES

### Patient 1

A 62-year-old male with end stage renal disease due to autosomal dominant polycystic kidney disease received a living unrelated donor kidney transplant in 2017 that was complicated by post-transplant lymphoproliferative disease involving the central nervous system, which developed in 2018. He was initiated on a course of intravenous (IV) rituximab and high-dose methotrexate. Due to new symptoms of fatigue and shortness of breath he was seen in his outpatient oncology clinic. There he described the fatigue to occur after walking four city blocks. The patient felt the symptoms were mild but did not recall having them three days prior. After discussing his concerns with his wife and oncologist, he was referred to the ED for workup of possible pulmonary embolism.

Physical exam revealed an anxious man with warm, well-perfused extremities and 2+/4 pitting edema in his right ankle. Upon questioning, he noted the swelling had developed after his last methotrexate infusion. Cardiac exam revealed a regular heart rate without third and fourth heart sounds. The neurological exam was non-focal. The patient had an eGFR of 48 milliliters (mL) per minute (min) per 1.73 squared meter (m^2^) (normal eGFR > 89 mL/min/m^2^). Fifth-generation serum troponin was not detectable. A chest radiograph revealed new trace bilateral pleural effusions and bibasilar atelectasis. Lower extremity Doppler ultrasonography showed fully compressible deep venous structures. Electrocardiograph showed normal sinus rhythm without axis deviation.

The patient was recommended to undergo computed tomographic angiography of the pulmonary arteries but was reluctant for concern that his scheduled chemotherapy the following week would be postponed due to a decline in kidney function following iodinated contrast exposure. Due to mild shortness of breath and no need for supplemental oxygen the patient was prepared to be discharged from the ED when a d-dimer level was reported to be 3254 nanograms (ng) per mL, which was 6.5-fold above the upper limit of the central laboratory normal range (0–500 ng/mL).

**Image 1:**
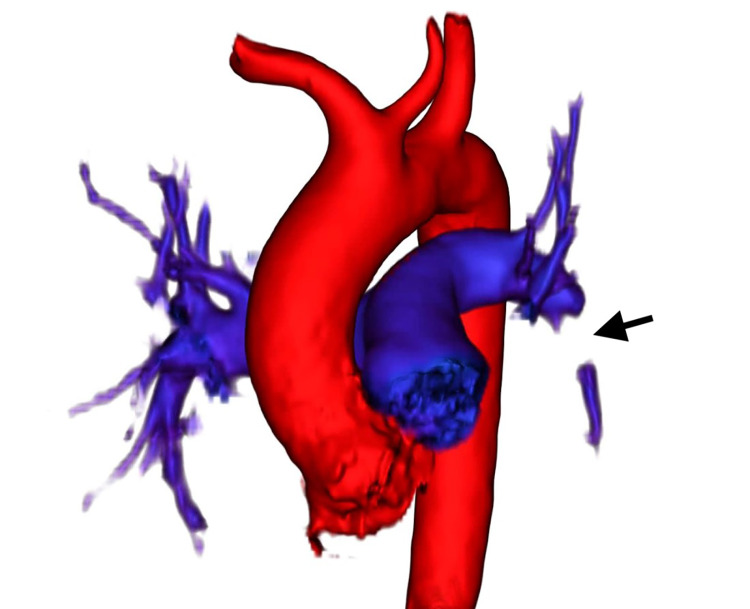
Patient 1, three-dimensional reconstruction of pulmonary embolus in left interlobar pulmonary embolus (anterior view) (black arrow) visualized by ferumoxytol-enhanced magnetic resonance angiography demonstrating total occlusion of the intravascular lumen.

**Image 2:**
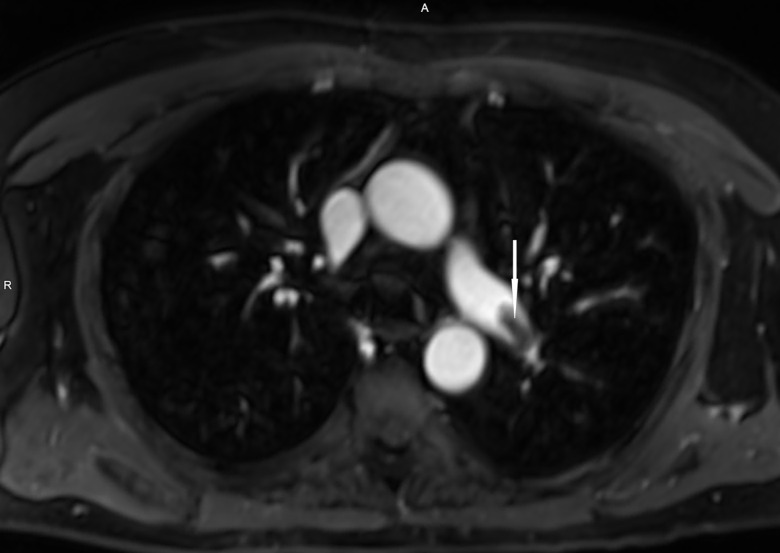
Patient 1, three-dimensional reconstruction of pulmonary embolus in left interlobar pulmonary embolus (axial view) (white arrow) visualized by ferumoxytol-enhanced magnetic resonance angiography demonstrating total occlusion of the intravascular lumen.

The renal transplant service was consulted for further recommendations. Considering his progressive shortness of breath, chronic kidney disease, malignancy, and elevated d-dimer, diagnostic imaging was considered. The patient was offered ferumoxytol-enhanced cardiothoracic angiography and counseled about risks and benefits of FeMRA as an alternative to iodinated contrast-enhanced radiologic imaging. The patient described neither an allergy to IV iron nor a history of iron deposition disease. The patient, his treating emergency physician, attending oncologist, attending transplant nephrologist, and attending radiologist were in agreement to use ferumoxytol off-label as a radiologic contrast agent. Therefore, IV ferumoxytol was then infused over 10 minutes with no evidence of anaphylaxis reported by the patient or observed by the nephrologist who was present during the duration of the infusion. The patient was required to lie flat. Images were acquired over a 20-minute period using a standard thoracic imaging protocol. FeMRA demonstrated a distal left main pulmonary artery embolus that extended to the left interlobar pulmonary artery (Images 1 and 2) without evidence of right heart strain by transthoracic echocardiography.

**CPC-EM Capsule**

What do we already know about this clinical entity?

Imaging plays a crucial role in the diagnosis and management of an acute pulmonary embolism, and often chest computed tomography angiogram or ventilation-perfusion scan is used.

What makes this presentation of disease reportable?

Ferumoxytol-enhanced magnetic resonance angiography (FeMRA) provided a rapid diagnosis of a life-threatening illness, which prompted the delivery of life-saving treatment.

What is the major learning point?

FeMRA of the cardiopulmonary vasculature can be used for the assessment of pulmonary embolism in patients with compromised renal function.

How might this improve emergency medicine practice?

This technique has the potential to be employed in emergency departments by using clinical resources already available.

The patient was admitted to the oncology service for initiation of anticoagulation with apixaban (10 mg tablet) to be given daily for one week and then 5 mg twice a day thereafter. The patient was discharged the following day and proceeded to outpatient chemotherapy infusion the following week. Four days afterward, non-contrasted magnetic resonance imaging (MRI) of the spine and brain was performed without gadolinium contrast. The repeat MRI occurred 93 hours after administration of ferumoxytol. There was no evidence of residual ferumoxytol obscuring the radiologist’s interpretation of the images.

### Patient 2

**Image 3:**
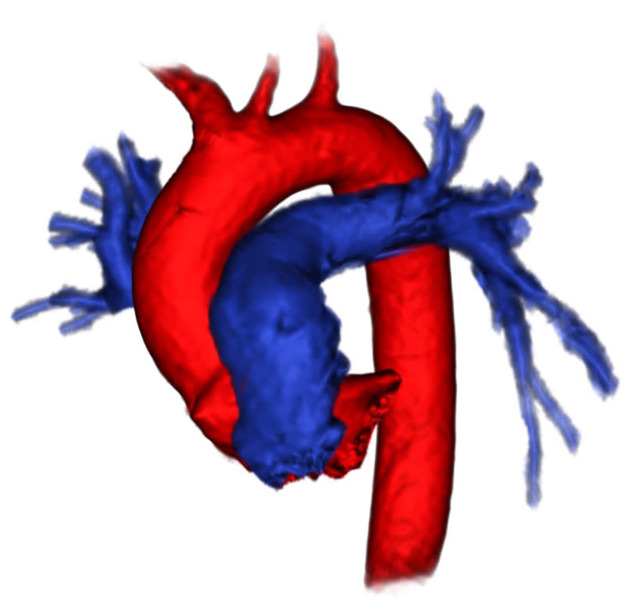
Patient 2, three-dimensional reconstruction of pulmonary arteries visualized by ferumoxytol-enhanced magnetic resonance angiography demonstrating widely patent intravascular lumens.

A 69-year-old male with CKD 5 (eGFR 11mL/min/1.73m^2^) was evaluated for kidney transplantation and utilized as a negative historical control. He had a history of pulmonary embolus 12 years prior without recurrence. At the time of transplant evaluation the patient was in his usual state of health. Due to a history of pulmonary embolus, coronary artery disease, CKD stage 5 and maintained urine output, FeMRA was performed. Images were acquired over a 20-minute period. Pulmonary vasculature was well visualized showing no defect of the lobar or interlobar pulmonary arteries. This patient demonstrated widely patent left and right pulmonary artery circulation with similar resolution compared to Patient #1 (Image 3). (All primary data is publicly available online: http://dx.doi.org/10.17632/s787bx8w52.2)

## DISCUSSION

Although, FeMRA of the abdominal vasculature has high diagnostic specificity and sensitivity,^10^ it has not been previously used to guide the treatment of acute pulmonary embolus. Ferumoxytol has also been used in the outpatient, inpatient, and ED settings for radiologic assessment of cardiothoracic pathology.^11^–^13^ Institutional safety protocols often require that a physician be present through the duration of each FeMRA procedure for concern of the theoretical risk of anaphylaxis. To avoid this reaction, infusion can be extended over a 10-minute period with a total dose based on ideal body weight of no more than 3 mg per kilogram (kg). Images can be fully acquired within 20 minutes of infusion, reducing the extended period of time often necessary to perform ventilation/perfusion scintigraphy. The rate of patient or healthcare staff reporting an allergic or anaphylactic reaction using the current protocol approximates 2%.^14^ The minimum adequate dose for clinically acceptable imaging is unknown for each anatomical site of interest. The current dosing regimen (3 mg/kg) is unlikely necessary for imaging of non-cardiothoracic vasculature in the abdomen where motion artifact can be readily attenuated. Such low-dose protocols may assist in further reducing the theoretical risk of iron overload.

Limitations of ferumoxytol use include active infection, iron deposition disease, and allergy to IV iron. Iron oxide facilitates bacterial growth and should not be used in patients suspected to have a diagnosis of sepsis. Iron deposition disease due to chronic IV iron use is well described. Finally, anaphylaxis with ferumoxytol has been reported to approach a rate of one event per 3000 patients infused with 510 mg of IV ferumoxytol over a 60-second period.^15^ The incidence of anaphylaxis using 10-minute infusion duration is unknown but anticipated to be less. Patients with prior allergy to IV iron, let alone anaphylaxis, should avoid ferumoxytol infusion.

The patient that we report here had a scheduled MRI study for re-staging of his malignancy four days after FeMRA and there was no evidence of retained ferumoxytol obscuring areas of the central nervous system where malignancy was first detected.

## CONCLUSION

Sequela of a pulmonary embolus is commonly encountered in the ED and can be life-threatening if not diagnosed and treated in a timely manner. Use of FeMRA represents a new tool that may offer expedited evaluation in patients with kidney dysfunction requiring intravascular contrast.
